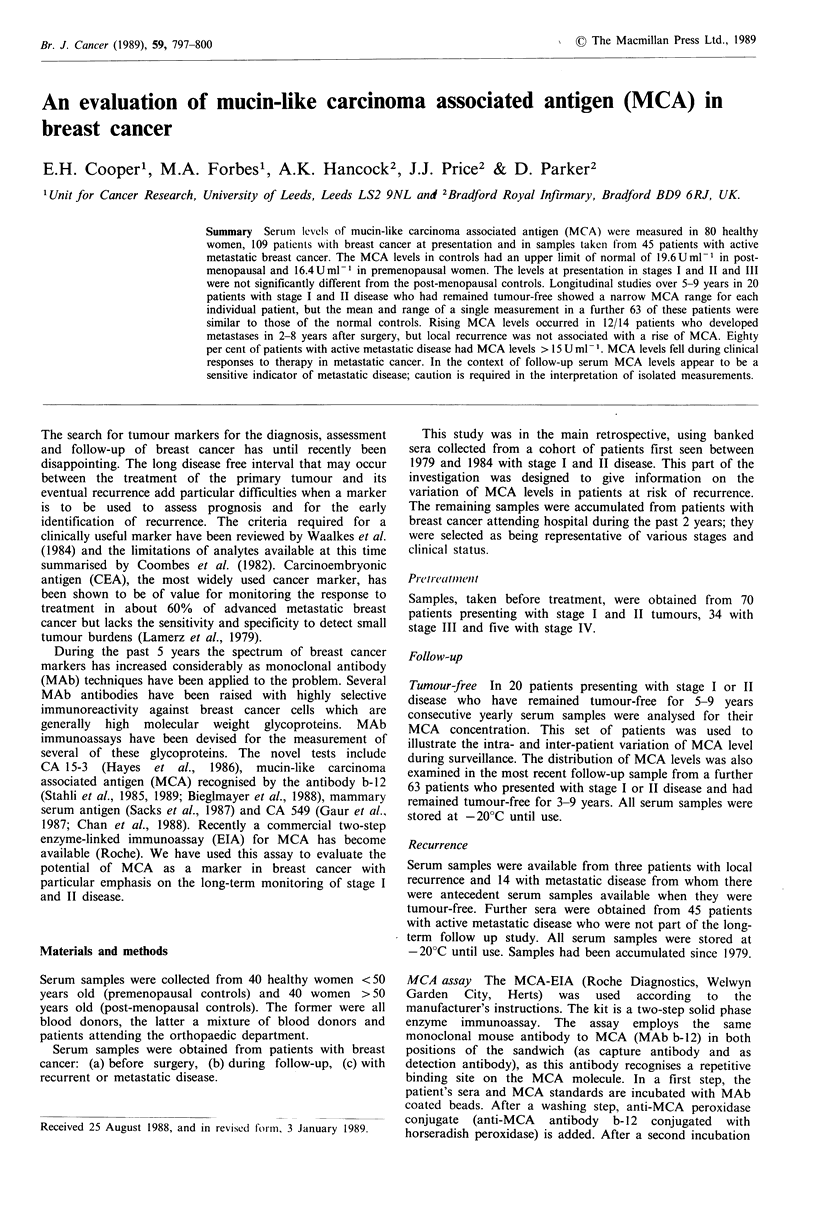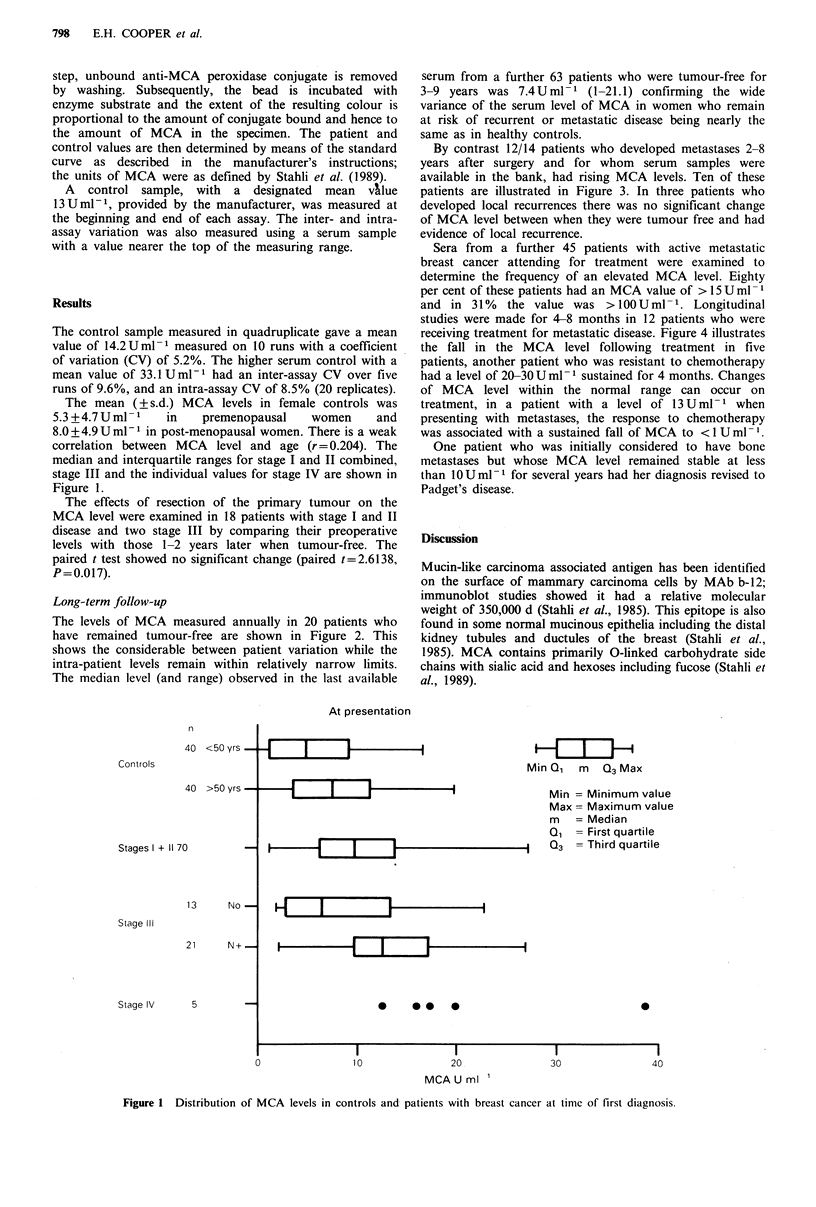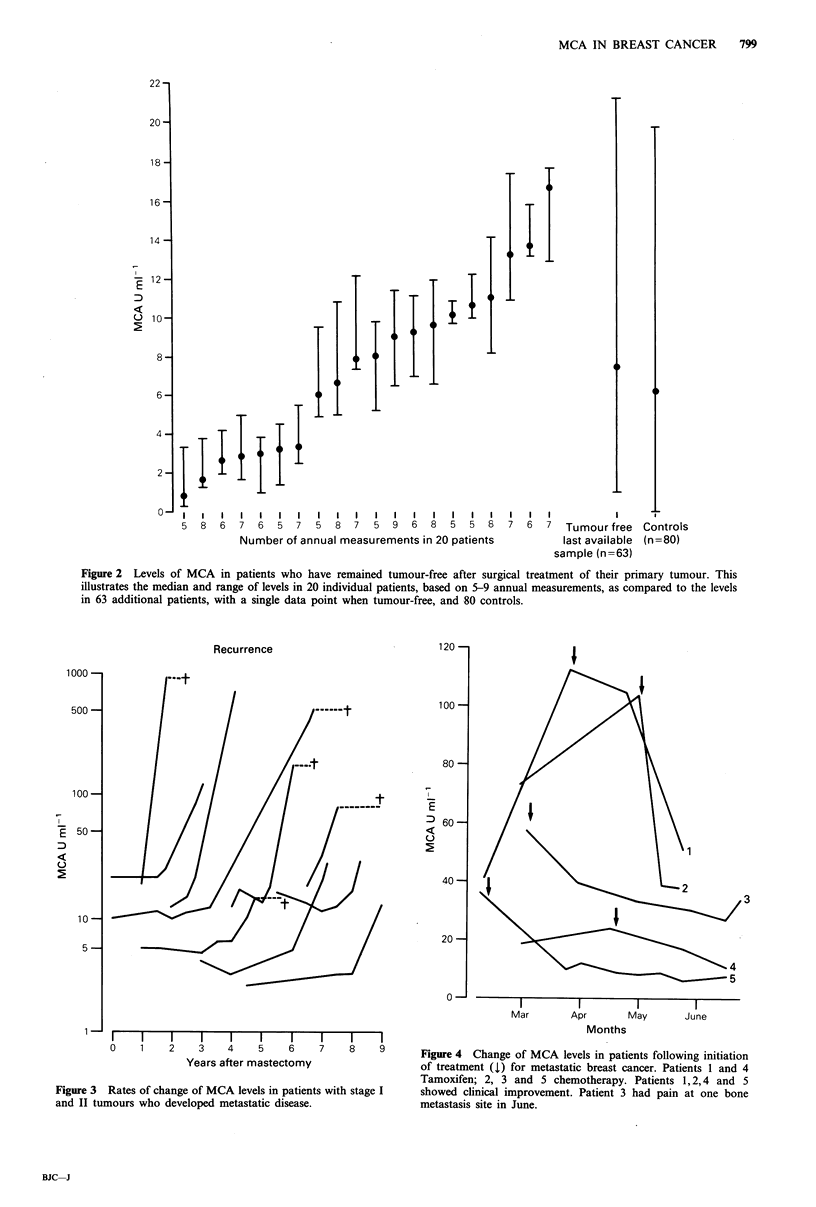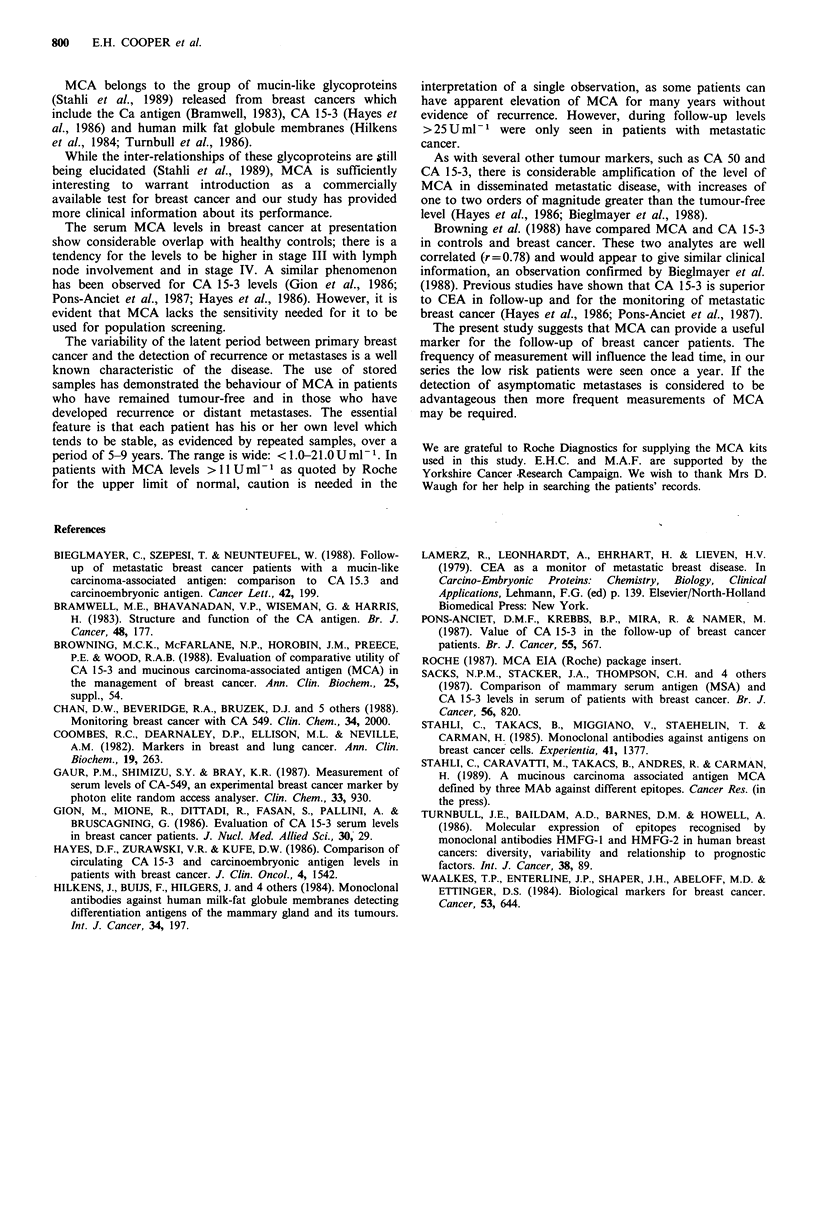# An evaluation of mucin-like carcinoma associated antigen (MCA) in breast cancer.

**DOI:** 10.1038/bjc.1989.166

**Published:** 1989-05

**Authors:** E. H. Cooper, M. A. Forbes, A. K. Hancock, J. J. Price, D. Parker

**Affiliations:** Unite for Cancer Research, University of Leeds, UK.

## Abstract

Serum levels of mucin-like carcinoma associated antigen (MCA) were measured in 80 healthy women, 109 patients with breast cancer at presentation and in samples taken from 45 patients with active metastatic breast cancer. The MCA levels in controls had an upper limit of normal of 19.6 U ml-1 in post-menopausal and 16.4 U ml-1 in premenopausal women. The levels at presentation in stages I and II and III were not significantly different from the post-menopausal controls. Longitudinal studies over 5-9 years in 20 patients with stage I and II disease who had remained tumour-free showed a narrow MCA range for each individual patient, but the mean and range of a single measurement in a further 63 of these patients were similar to those of the normal controls. Rising MCA levels occurred in 12/14 patients who developed metastases in 2-8 years after surgery, but local recurrence was not associated with a rise of MCA. Eighty per cent of patients with active metastatic disease had MCA levels greater than 15 U ml-1. MCA levels fell during clinical responses to therapy in metastatic cancer. In the context of follow-up serum MCA levels appear to be a sensitive indicator of metastatic disease; caution is required in the interpretation of isolated measurements.


Br. J. Cancer (1989), 59, 797–800 © The Macmillan Press Ltd., 1989

### An evaluation of mucin-like carcinoma associated antigen (MCA) in breast cancer

E.H. Cooper[1], M.A. Forbes[1], A.K. Hancock[2], J.J. Price[2] & D. Parker[2]

[1]*Unit for Cancer Research, University of Leeds, Leeds LS2 9NL and* [2]*Bradford Royal Infirmary, Bradford BD9 6RJ, UK.*

**Summary** Serum levels of mucin-like carcinoma associated antigen (MCA) were measured in 80 healthy women, 109 patients with breast cancer at presentation and in samples taken from 45 patients with active metastatic breast cancer. The MCA levels in controls had an upper limit of normal of 19.6 U ml$^{-1}$ in post-menopausal and 16.4 U ml$^{-1}$ in premenopausal women. The levels at presentation in stages I and II and III were not significantly different from the post-menopausal controls. Longitudinal studies over 5–9 years in 20 patients with stage I and II disease who had remained tumour-free showed a narrow MCA range for each individual patient, but the mean and range of a single measurement in a further 63 of these patients were similar to those of the normal controls. Rising MCA levels occurred in 12/14 patients who developed metastases in 2–8 years after surgery, but local recurrence was not associated with a rise of MCA. Eighty per cent of patients with active metastatic disease had MCA levels $>15$ U ml$^{-1}$. MCA levels fell during clinical responses to therapy in metastatic cancer. In the context of follow-up serum MCA levels appear to be a sensitive indicator of metastatic disease; caution is required in the interpretation of isolated measurements.

The search for tumour markers for the diagnosis, assessment and follow-up of breast cancer has until recently been disappointing. The long disease free interval that may occur between the treatment of the primary tumour and its eventual recurrence add particular difficulties when a marker is to be used to assess prognosis and for the early identification of recurrence. The criteria required for a clinically useful marker have been reviewed by Waalkes *et al.* (1984) and the limitations of analytes available at this time summarised by Coombes *et al.* (1982). Carcinoembryonic antigen (CEA), the most widely used cancer marker, has been shown to be of value for monitoring the response to treatment in about 60% of advanced metastatic breast cancer but lacks the sensitivity and specificity to detect small tumour burdens (Lamerz *et al.*, 1979).

During the past 5 years the spectrum of breast cancer markers has increased considerably as monoclonal antibody (MAb) techniques have been applied to the problem. Several MAb antibodies have been raised with highly selective immunoreactivity against breast cancer cells which are generally high molecular weight glycoproteins. MAb immunoassays have been devised for the measurement of several of these glycoproteins. The novel tests include CA 15-3 (Hayes *et al.*, 1986), mucin-like carcinoma associated antigen (MCA) recognised by the antibody b-12 (Stahli *et al.*, 1985, 1989; Bieglmayer *et al.*, 1988), mammary serum antigen (Sacks *et al.*, 1987) and CA 549 (Gaur *et al.*, 1987; Chan *et al.*, 1988). Recently a commercial two-step enzyme-linked immunoassay (EIA) for MCA has become available (Roche). We have used this assay to evaluate the potential of MCA as a marker in breast cancer with particular emphasis on the long-term monitoring of stage I and II disease.

### Materials and methods

Serum samples were collected from 40 healthy women $<50$ years old (premenopausal controls) and 40 women $>50$ years old (post-menopausal controls). The former were all blood donors, the latter a mixture of blood donors and patients attending the orthopaedic department.

Serum samples were obtained from patients with breast cancer: (a) before surgery, (b) during follow-up, (c) with recurrent or metastatic disease.

Received 25 August 1988, and in revised form, 3 January 1989.

This study was in the main retrospective, using banked sera collected from a cohort of patients first seen between 1979 and 1984 with stage I and II disease. This part of the investigation was designed to give information on the variation of MCA levels in patients at risk of recurrence. The remaining samples were accumulated from patients with breast cancer attending hospital during the past 2 years; they were selected as being representative of various stages and clinical status.

### *Pretreatment*

Samples, taken before treatment, were obtained from 70 patients presenting with stage I and II tumours, 34 with stage III and five with stage IV.

### *Follow-up*

*Tumour-free* In 20 patients presenting with stage I or II disease who have remained tumour-free for 5–9 years consecutive yearly serum samples were analysed for their MCA concentration. This set of patients was used to illustrate the intra- and inter-patient variation of MCA level during surveillance. The distribution of MCA levels was also examined in the most recent follow-up sample from a further 63 patients who presented with stage I or II disease and had remained tumour-free for 3–9 years. All serum samples were stored at $-20°C$ until use.

### *Recurrence*

Serum samples were available from three patients with local recurrence and 14 with metastatic disease from whom there were antecedent serum samples available when they were tumour-free. Further sera were obtained from 45 patients with active metastatic disease who were not part of the long-term follow up study. All serum samples were stored at $-20°C$ until use. Samples had been accumulated since 1979.

*MCA assay* The MCA-EIA (Roche Diagnostics, Welwyn Garden City, Herts) was used according to the manufacturer's instructions. The kit is a two-step solid phase enzyme immunoassay. The assay employs the same monoclonal mouse antibody to MCA (MAb b-12) in both positions of the sandwich (as capture antibody and as detection antibody), as this antibody recognises a repetitive binding site on the MCA molecule. In a first step, the patient's sera and MCA standards are incubated with MAb coated beads. After a washing step, anti-MCA peroxidase conjugate (anti-MCA antibody b-12 conjugated with horseradish peroxidase) is added. After a second incubation step, unbound anti-MCA peroxidase conjugate is removed by washing. Subsequently, the bead is incubated with enzyme substrate and the extent of the resulting colour is proportional to the amount of conjugate bound and hence to the amount of MCA in the specimen. The patient and control values are then determined by means of the standard curve as described in the manufacturer's instructions; the units of MCA were as defined by Stahli *et al.* (1989).

A control sample, with a designated mean value $13\,U\,ml^{-1}$, provided by the manufacturer, was measured at the beginning and end of each assay. The inter- and intra-assay variation was also measured using a serum sample with a value nearer the top of the measuring range.

### Results

The control sample measured in quadruplicate gave a mean value of $14.2\,U\,ml^{-1}$ measured on 10 runs with a coefficient of variation (CV) of 5.2%. The higher serum control with a mean value of $33.1\,U\,ml^{-1}$ had an inter-assay CV over five runs of 9.6%, and an intra-assay CV of 8.5% (20 replicates).

The mean ($\pm$s.d.) MCA levels in female controls was $5.3\pm4.7\,U\,ml^{-1}$ in premenopausal women and $8.0\pm4.9\,U\,ml^{-1}$ in post-menopausal women. There is a weak correlation between MCA level and age ($r=0.204$). The median and interquartile ranges for stage I and II combined, stage III and the individual values for stage IV are shown in Figure 1.

The effects of resection of the primary tumour on the MCA level were examined in 18 patients with stage I and II disease and two stage III by comparing their preoperative levels with those 1–2 years later when tumour-free. The paired $t$ test showed no significant change (paired $t=2.6138$, $P=0.017$).

### *Long-term follow-up*

The levels of MCA measured annually in 20 patients who have remained tumour-free are shown in Figure 2. This shows the considerable between patient variation while the intra-patient levels remain within relatively narrow limits. The median level (and range) observed in the last available serum from a further 63 patients who were tumour-free for 3–9 years was $7.4\,U\,ml^{-1}$ (1–21.1) confirming the wide variance of the serum level of MCA in women who remain at risk of recurrent or metastatic disease being nearly the same as in healthy controls.

By contrast 12/14 patients who developed metastases 2–8 years after surgery and for whom serum samples were available in the bank, had rising MCA levels. Ten of these patients are illustrated in Figure 3. In three patients who developed local recurrences there was no significant change of MCA level between when they were tumour free and had evidence of local recurrence.

Sera from a further 45 patients with active metastatic breast cancer attending for treatment were examined to determine the frequency of an elevated MCA level. Eighty per cent of these patients had an MCA value of $>15\,U\,ml^{-1}$ and in 31% the value was $>100\,U\,ml^{-1}$. Longitudinal studies were made for 4–8 months in 12 patients who were receiving treatment for metastatic disease. Figure 4 illustrates the fall in the MCA level following treatment in five patients, another patient who was resistant to chemotherapy had a level of $20–30\,U\,ml^{-1}$ sustained for 4 months. Changes of MCA level within the normal range can occur on treatment, in a patient with a level of $13\,U\,ml^{-1}$ when presenting with metastases, the response to chemotherapy was associated with a sustained fall of MCA to $<1\,U\,ml^{-1}$.

One patient who was initially considered to have bone metastases but whose MCA level remained stable at less than $10\,U\,ml^{-1}$ for several years had her diagnosis revised to Padget's disease.

### Discussion

Mucin-like carcinoma associated antigen has been identified on the surface of mammary carcinoma cells by MAb b-12; immunoblot studies showed it had a relative molecular weight of 350,000 d (Stahli *et al.*, 1985). This epitope is also found in some normal mucinous epithelia including the distal kidney tubules and ductules of the breast (Stahli *et al.*, 1985). MCA contains primarily O-linked carbohydrate side chains with sialic acid and hexoses including fucose (Stahli *et al.*, 1989).

**Figure 1** Distribution of MCA levels in controls and patients with breast cancer at time of first diagnosis.

**Figure 2** Levels of MCA in patients who have remained tumour-free after surgical treatment of their primary tumour. This illustrates the median and range of levels in 20 individual patients, based on 5–9 annual measurements, as compared to the levels in 63 additional patients, with a single data point when tumour-free, and 80 controls.

**Figure 3** Rates of change of MCA levels in patients with stage I and II tumours who developed metastatic disease.

**Figure 4** Change of MCA levels in patients following initiation of treatment (↓) for metastatic breast cancer. Patients 1 and 4 Tamoxifen; 2, 3 and 5 chemotherapy. Patients 1, 2, 4 and 5 showed clinical improvement. Patient 3 had pain at one bone metastasis site in June.

MCA belongs to the group of mucin-like glycoproteins (Stahli *et al.*, 1989) released from breast cancers which include the Ca antigen (Bramwell, 1983), CA 15-3 (Hayes *et al.*, 1986) and human milk fat globule membranes (Hilkens *et al.*, 1984; Turnbull *et al.*, 1986).

While the inter-relationships of these glycoproteins are still being elucidated (Stahli *et al.*, 1989), MCA is sufficiently interesting to warrant introduction as a commercially available test for breast cancer and our study has provided more clinical information about its performance.

The serum MCA levels in breast cancer at presentation show considerable overlap with healthy controls; there is a tendency for the levels to be higher in stage III with lymph node involvement and in stage IV. A similar phenomenon has been observed for CA 15-3 levels (Gion *et al.*, 1986; Pons-Anciet *et al.*, 1987; Hayes *et al.*, 1986). However, it is evident that MCA lacks the sensitivity needed for it to be used for population screening.

The variability of the latent period between primary breast cancer and the detection of recurrence or metastases is a well known characteristic of the disease. The use of stored samples has demonstrated the behaviour of MCA in patients who have remained tumour-free and in those who have developed recurrence or distant metastases. The essential feature is that each patient has his or her own level which tends to be stable, as evidenced by repeated samples, over a period of 5–9 years. The range is wide: $<1.0$–$21.0\,U\,ml^{-1}$. In patients with MCA levels $>11\,U\,ml^{-1}$ as quoted by Roche for the upper limit of normal, caution is needed in the interpretation of a single observation, as some patients can have apparent elevation of MCA for many years without evidence of recurrence. However, during follow-up levels $>25\,U\,ml^{-1}$ were only seen in patients with metastatic cancer.

As with several other tumour markers, such as CA 50 and CA 15-3, there is considerable amplification of the level of MCA in disseminated metastatic disease, with increases of one to two orders of magnitude greater than the tumour-free level (Hayes *et al.*, 1986; Bieglmayer *et al.*, 1988).

Browning *et al.* (1988) have compared MCA and CA 15-3 in controls and breast cancer. These two analytes are well correlated ($r=0.78$) and would appear to give similar clinical information, an observation confirmed by Bieglmayer *et al.* (1988). Previous studies have shown that CA 15-3 is superior to CEA in follow-up and for the monitoring of metastatic breast cancer (Hayes *et al.*, 1986; Pons-Anciet *et al.*, 1987).

The present study suggests that MCA can provide a useful marker for the follow-up of breast cancer patients. The frequency of measurement will influence the lead time, in our series the low risk patients were seen once a year. If the detection of asymptomatic metastases is considered to be advantageous then more frequent measurements of MCA may be required.

We are grateful to Roche Diagnostics for supplying the MCA kits used in this study. E.H.C. and M.A.F. are supported by the Yorkshire Cancer Research Campaign. We wish to thank Mrs D. Waugh for her help in searching the patients' records.

### References

BIEGLMAYER, C., SZEPESI, T. & NEUNTEUFEL, W. (1988). Follow-up of metastatic breast cancer patients with a mucin-like carcinoma-associated antigen: comparison to CA 15.3 and carcinoembryonic antigen. *Cancer Lett.*, **42**, 199.

BRAMWELL, M.E., BHAVANADAN, V.P., WISEMAN, G. & HARRIS, H. (1983). Structure and function of the CA antigen. *Br. J. Cancer*, **48**, 177.

BROWNING, M.C.K., McFARLANE, N.P., HOROBIN, J.M., PREECE, P.E. & WOOD, R.A.B. (1988). Evaluation of comparative utility of CA 15-3 and mucinous carcinoma-associated antigen (MCA) in the management of breast cancer. *Ann. Clin. Biochem.*, **25**, suppl., 54.

CHAN, D.W., BEVERIDGE, R.A., BRUZEK, D.J. and 5 others (1988). Monitoring breast cancer with CA 549. *Clin. Chem.*, **34**, 2000.

COOMBES, R.C., DEARNALEY, D.P., ELLISON, M.L. & NEVILLE, A.M. (1982). Markers in breast and lung cancer. *Ann. Clin. Biochem.*, **19**, 263.

GAUR, P.M., SHIMIZU, S.Y. & BRAY, K.R. (1987). Measurement of serum levels of CA-549, an experimental breast cancer marker by photon elite random access analyser. *Clin. Chem.*, **33**, 930.

GION, M., MIONE, R., DITTADI, R., FASAN, S., PALLINI, A. & BRUSCAGNING, G. (1986). Evaluation of CA 15-3 serum levels in breast cancer patients. *J. Nucl. Med. Allied Sci.*, **30**, 29.

HAYES, D.F., ZURAWSKI, V.R. & KUFE, D.W. (1986). Comparison of circulating CA 15-3 and carcinoembryonic antigen levels in patients with breast cancer. *J. Clin. Oncol.*, **4**, 1542.

HILKENS, J., BUIJS, F., HILGERS, J. and 4 others (1984). Monoclonal antibodies against human milk-fat globule membranes detecting differentiation antigens of the mammary gland and its tumours. *Int. J. Cancer*, **34**, 197.

LAMERZ, R., LEONHARDT, A., EHRHART, H. & LIEVEN, H.V. (1979). CEA as a monitor of metastatic breast disease. In *Carcino-Embryonic Proteins: Chemistry, Biology, Clinical Applications*, Lehmann, F.G. (ed) p. 139. Elsevier/North-Holland Biomedical Press: New York.

PONS-ANCIET, D.M.F., KREBBS, B.P., MIRA, R. & NAMER, M. (1987). Value of CA 15-3 in the follow-up of breast cancer patients. *Br. J. Cancer*, **55**, 567.

ROCHE (1987). MCA EIA (Roche) package insert.

SACKS, N.P.M., STACKER, J.A., THOMPSON, C.H. and 4 others (1987). Comparison of mammary serum antigen (MSA) and CA 15-3 levels in serum of patients with breast cancer. *Br. J. Cancer*, **56**, 820.

STAHLI, C., TAKACS, B., MIGGIANO, V., STAEHELIN, T. & CARMAN, H. (1985). Monoclonal antibodies against antigens on breast cancer cells. *Experientia*, **41**, 1377.

STAHLI, C., CARAVATTI, M., TAKACS, B., ANDRES, R. & CARMAN, H. (1989). A mucinous carcinoma associated antigen MCA defined by three MAb against different epitopes. *Cancer Res.* (in the press).

TURNBULL, J.E., BAILDAM, A.D., BARNES, D.M. & HOWELL, A. (1986). Molecular expression of epitopes recognised by monoclonal antibodies HMFG-1 and HMFG-2 in human breast cancers: diversity, variability and relationship to prognostic factors. *Int. J. Cancer*, **38**, 89.

WAALKES, T.P., ENTERLINE, J.P., SHAPER, J.H., ABELOFF, M.D. & ETTINGER, D.S. (1984). Biological markers for breast cancer. *Cancer*, **53**, 644.